# Author Correction: The mediating role of resilience in the effects of physical exercise on college students’ negative emotions during the COVID-19 epidemic

**DOI:** 10.1038/s41598-022-05430-5

**Published:** 2022-01-17

**Authors:** Xuening Li, Huasen Yu, Ning Yang

**Affiliations:** 1 CNRS, Brain and Cognition Research Center (CerCo), Université de Toulouse 3, Toulouse, France; 2 College of Physical Education and Health, East China Normal University, Shanghai, China; 3 Institute of Physical Education, Shandong Youth University of Political Science, Jinan, China

Correction to: *Scientific Reports* https://doi.org/10.1038/s41598-021-04336-y, published online 31 December 2021

The original version of this Article contained an error in Reference 29, which was incorrectly given as:

Chen, P. *et al*. Wuhan coronavirus (2019-nCoV): The need to maintain regular physical activity while taking precautions. *J. Sport Health Sci.* **9**, 103 (2020).

The correct reference is listed below:

Chen, P. *et al*. Coronavirus disease (COVID-19): The need to maintain regular physical activity while taking precautions. *J. Sport Health Sci.*. **9**, 103–104 (2020).

The original Article has been corrected.

